# Shared Learning Utilizing Digital Methods in Surgery to Enhance Transparency in Surgical Innovation: Protocol for a Scoping Review

**DOI:** 10.2196/37544

**Published:** 2022-09-08

**Authors:** Christin Hoffmann, Matthew Kobetic, Natasha Alford, Natalie Blencowe, Jozel Ramirez, Rhiannon Macefield, Jane M Blazeby, Kerry N L Avery, Shelley Potter

**Affiliations:** 1 National Institute for Health and Care Research, Bristol Biomedical Research Centre Population Health Sciences, Bristol Medical School University of Bristol Bristol United Kingdom; 2 Division of Surgery Bristol Royal Infirmary University Hospitals Bristol and Weston National Health Service Foundation Trust Bristol United Kingdom; 3 Bristol Breast Care Centre North Bristol National Health Service Trust Bristol United Kingdom; 4 Translational Health Sciences Bristol Medical School University of Bristol Bristol United Kingdom

**Keywords:** innovation, surgery, surgical, shared learning, scoping review, operative, procedures, digital, training, learning, feedback, digital method, review, review methodology, surgeon, education, medical education, eHealth, digital health, digital tool

## Abstract

**Background:**

Surgical innovation can lead to important improvements in patient outcomes. Currently, information and knowledge about novel procedures and devices are disseminated informally and in an unstandardized way (eg, through social media). This can lead to ineffective and inefficient knowledge sharing among surgeons, which can result in the harmful repetition of mistakes and delay in the uptake of promising innovation. Improvements are needed in the way that learning in surgical innovation is shared through the development of novel, real-time methods, informed by a contemporary and comprehensive investigation of existing methods.

**Objective:**

The aim of this scoping review is to explore the application of existing digital methods for training/education and feedback to surgeons in the context of performing invasive surgical procedures. This work will (1) summarize existing methods for shared learning in surgery and how they are characterized and operationalized, (2) examine the impact of their application, and (3) explore their benefits and barriers to implementation. The findings of this scoping review will inform the development of novel, real-time methods to optimize shared learning in surgical innovation.

**Methods:**

This study will adhere to the recommended guidelines for conducting scoping reviews. A total of 6 different searches will be conducted within multiple sources (2 electronic databases, journals, social media, gray literature, commercial websites, and snowball searches) to comprehensively identify relevant articles and data. Searches will be limited to articles published in the English language within the last 5 years. Wherever possible, a 2-stage study selection process will be followed whereby the eligibility of articles will be assessed through the title, abstract, and full-text screening independently by 2 reviewers. Inclusion criteria will be articles providing data on (1) fully qualified theater staff involved in performing invasive procedures, (2) one or more methods for shared learning (ie, digital means for training/education and feedback), and (3) qualitative or quantitative evaluations of this method. Data will be extracted (10% double data extraction by an independent reviewer) into a piloted proforma and analyzed using descriptive statistics, narrative summaries, and principles of thematic analysis.

**Results:**

The study commenced in October 2021 and is planned to be completed in 2023. To date, systematic searches were applied to 2 electronic databases (MEDLINE and Web of Science) and returned a total of 10,093 records. The results of this scoping review will be published as open access in a peer-reviewed journal.

**Conclusions:**

This scoping review of methods for shared learning in surgery is, to our knowledge, the most comprehensive and up-to-date investigation that maps current information on this topic. Ultimately, efficient and effective sharing of information and knowledge of novel procedures and devices has the potential to optimize the evaluation of early-phase surgical research and reduce harmful innovation.

**International Registered Report Identifier (IRRID):**

PRR1-10.2196/37544

## Introduction

### Background

Surgical innovation is common and plays a crucial role in advancing surgical practice. It is characterized by a developmental process whereby novel procedures and devices evolve from early ideas and first-in-human studies to longer-term evaluations. Surgeons acquire important learning from incremental cases [1-3], leading to a steep learning curve in the early phases of technique development. Transparent sharing of case-by-case learning is therefore critical to promote efficient and safe innovation and timely evaluation of surgical innovation when a new technique has stabilized [2,4].

Currently, surgeons tend to innovate independently. Early incremental learning, including modifications to the technique and its outcomes, is rarely shared beyond the local team, if at all. Traditionally, dissemination of information about innovative procedures occurs at a relatively late stage through surgeon innovators presenting their technique at meetings and conferences, followed by taught courses and peer-reviewed publications [1,5]. Key incremental case-by-case learning is often not recorded. Evidence in other areas has shown that the outcomes of using an innovation are positively affected when knowledge is shared between external stakeholders [6], which can even be a source of innovation itself [7]. Increased shared learning in surgical innovation may provide similar benefits.

Disseminating new knowledge in health care is known to be challenging [8]. The process of exchanging information can be influenced by a multiplicity of factors, including organizational, cultural, social, and psychological influences [9,10] and facilitated by technology [11]. More recently, surgeon innovators are increasingly utilizing digital platforms and social media to disseminate ideas and practice [12]. While this has notable benefits, the acquired knowledge is shared inconsistently [13,14]. It may promote optimism bias by preferentially favoring positive developments and outcomes and is unsuited to building a robust evidence base [15]. Furthermore, approaches to disseminating information provide little or no scope for feedback, hindering efficient innovation that can address learning curve effects. This may also mean the benefits and harm outcomes of innovation are underreported or not shared transparently, and opportunities to promote patient safety by avoiding repetition of potentially harmful mistakes are therefore lost. Methods for effectively and transparently sharing information in real time are needed to accommodate incremental, case-by-case learning when developing a new surgical technique. Such methods must also include mechanisms for the confidential provision of feedback to avoid patient harm while simultaneously ensuring a safe space for surgical innovation.

A number of digital methods to provide feedback exist, including image analysis [16], artificial intelligence [17], or virtual and telementoring platforms [18]. These have been demonstrated to improve outcomes relevant to patients (eg, reduced operative time) and surgeons (eg, improved surgical skills) for established procedures [19,20]. It is therefore possible that similar methods could be used or adapted to capture the incremental learning associated with an innovative surgical technique in near real time. Digital methods may also enable prompt sharing to facilitate efficient, transparent, and safe innovation of novel surgical procedures.

There is no standard definition of surgical innovation, and descriptions of novelty vary considerably across the literature [21,22]. Identification of relevant literature on surgical innovation is also hindered by poor reporting [4,23,24]. Innovations are “frequently reported as information communications which may not be well organized and are sometimes anecdotal“ (pg 1) [25]. Standard systematic review methodology would therefore be unlikely to identify relevant studies consistently and reliably. A literature synthesis that adopts a broad approach to include a wide variety of publication types is required to capture a range of digital methods for shared learning in surgery in general. Initial scoping searches (using Google) showed no such review has been conducted. A scoping review is considered a suitable approach for mapping a complex topic area where no prior investigation exists [26-28]. Methods allow inclusion of a range of study designs without requiring a formal quality assessment of the included articles. A scoping review of currently available methods used to share learning in surgery can identify potentially relevant digital methods, which in turn can inform the development of novel methods to optimize shared learning in surgical innovation.

### Aims and Objectives

The aim of this scoping review is to explore the application of digital methods for training/education and feedback for shared learning in the context of invasive surgical procedures. We aimed to:

1. Summarize existing methods for shared learning (ie, digital methods for learning or education *and* feedback) and how they are characterized and operationalized
2. Examine the impact of the applications of methods for shared learning from data on the evaluation of methods
3. Explore benefits and barriers to the implementation of methods for shared learning from data on the evaluation of methods

Results will inform strategies for embedding suitable methods within an electronic platform for real-time reporting and sharing of outcomes of surgical innovation.

## Methods

### Overview

A scoping review was chosen to investigate this topic due to the breadth and type of the data of interest for the research question. This scoping review will be conducted adhering to the PRISMA (Preferred Reporting Items for Systematic Reviews and Meta-Analyses) extension for scoping reviews (PRISMA-ScR) and established frameworks for conducting scoping reviews [26,28-30]. An initially completed checklist can be found in Multimedia Appendix 1 [28], and an updated checklist will be provided upon publication of results. Two trained reviewers will conduct the review, with input from a multidisciplinary team consisting of surgeons, methodologists, health services researchers, and social scientists. Any necessary deviations from the current protocol will be reported in the completed manuscript.

### Ethical Considerations

Ethical approval for this scoping review was not required, because it does not involve human participants, their tissue, data or samples or has ethical implications, as outlined in institutional policies including Section 2 of the University of Bristol's Ethics of Research Policy and Procedure.

### Definitions

#### Shared Learning

There is no consensus definition of shared learning in the health care or surgical literature [31]. For the purposes of this review, we have defined shared learning as a method of providing training/education with feedback to 2 or more clinicians undertaking surgical procedures. Examples of training or education and feedback in surgery can include but are not limited to assessment of skills; performance or outcomes; proctorship, mentoring, apprenticeships; and demonstration of techniques and simulation.

#### Digital Methods

Digital methods will be defined as utilizing electronic technology that is able to generate, store, and process data. In this review, we will exclude any methods that are used in situ (eg, laparoscopic equipment that includes cameras to broadcast to screens located in the operating theater).

#### Invasive Procedure

An invasive procedure is defined as “one where purposeful/deliberate access to the body is gained via an incision, percutaneous puncture, where instrumentation is used in addition to the puncture needle, or instrumentation via a natural orifice. It begins when entry to the body is gained and ends when the instrument is removed, and/or the skin is closed. Invasive procedures are performed by trained healthcare professionals using instruments, which include, but are not limited to, endoscopes, catheters, scalpels, scissors, devices and tubes” (pg 2) [32].

#### Surgical Innovation

There is no agreed definition of innovative surgical innovation [21,22,33] and no validated methods to identify the phase of evaluation retrospectively in the published literature. Innovative surgical procedures were therefore defined as those where authors self-report an invasive procedure as “new” or “modified,” corresponding to phases 1, 2a, and 2b of the IDEAL (Idea, Development, Exploration, Assessment, Long-term study) framework [34,35].

### Identifying Relevant Articles

#### Data Sources

Scoping searches conducted to inform our study have confirmed that relevant information is contained in a variety of data sources beyond traditional peer-reviewed publications. A number of different data sources are proposed to be of value for navigating the unique landscape of available evidence and addressing the study aim (Table 1).

**Table 1:** Publication types considered in this review, with examples of possible data sources.

Publication type	Possible data sources (examples)
Peer-reviewed publications	Protocols, conference abstracts, empirical studies of any publication type (eg, pilot, feasibility, methodological, diagnostic accuracy, intervention, and observational studies)
Opinion pieces	Editorials, comments, letters, perspectives, news, bulletins
Social media	Twitter posts, YouTube videos
Gray literature	Scientific, academic, government, or commercial reports (eg, reports of artificial intelligence or virtual reality systems)
Commercial online resources	Websites of manufacturers and platform/software/hardware providers related to training/education and feedback systems (eg, Johnson & Johnson, Medtronic, Proximie, Explorer Surgical, Visual Lab 360, Kognito, Oxford Medical Simulation, and Immersive Touch)

### Searches

A total of 6 approaches will be followed to identify relevant data sources detailed above. Collaboration with a subject librarian will aid optimization of searches and inclusivity of search terms throughout.

#### Electronic Database Searches

A comprehensive search strategy for conducting electronic database searches will be developed. Keywords will be based on the study eligibility criteria using the search strings “shared learning” AND “methods” AND “invasive procedures.” Targeted internet searches and relevant existing search strategies (eg, for invasive procedures [32]) will be used to inform the list of keywords. Search strategies can be found in Multimedia Appendix 2.

The search strategy will be translated to search for relevant publications in MEDLINE (Ovid version) and Web of Science.

#### Journal Searches

Scientific journals that are likely to publish relevant papers will be searched manually to identify any peer-reviewed articles that may be missed through electronic database searches. Contents pages of journals will be reviewed with a date of publication within the last 5 years. Journals of interest will be identified through expert knowledge and journal databases (eg, Web of Science Master Journal List). Relevant journals identified a priori include *Journal of Medical Internet Research*, *BMJ Surgery*, *Interventions & Health Technologies*, *Surgical Innovation*, *Health Information Research*, *Methods of Information in Medicine*, and *Applied Clinical Informatics*. Additional journals from previously identified articles will be added as appropriate.

#### Social Media

Social media platforms Twitter and YouTube have been identified as common sources for sharing knowledge about surgical innovation [36] and will therefore serve as an additional data source to inform the extent of their utility. Multiple different methods for querying social media platforms exist with known advantages and limitations [37]. Information will be searched by entering keywords related to “surgery” (eg, surgical, procedure) and “innovation” (eg, novel, improved, recently developed, adapted) into the social media platforms’ own advanced search functions (eg, Twitter application programming interface). These functions are free to use, providing access to 1% of real-time content. Automated dashboard vendors provide licensed software for the retrieval and analysis of social media content (eg, Mediatoolkit, Radian6). Dashboard vendors provide access to the full content of posts across a range of social media platforms. They will be considered to supplement searches if social media posts identified through advanced search functions are considered insufficient. The same keywords will be used and combined with Boolean search operators to retrieve relevant content.

#### Gray Literature

A search of the gray literature will be conducted to identify potentially relevant articles not indexed in electronic databases. Specific sources to search for gray literature include OpenGrey, Canada’s Drug and Health Technology Agency's Gray Matters, Healthcare Management Information Consortium, National Technical Information Service, and American Psychological Association PsycExtra, and internet searches (eg, using Google). Simple search terms will be used for these searches and adjusted based on gray literature sources and results. Any adjustments, if necessary, will be reported in the final manuscript.

#### Handsearching of Commercial Websites

Websites of commercial providers of digital platforms or software that are known to the research team will be searched to identify further relevant information on digital methods for shared learning. Relevant websites of known surgical technologies and technology providers will include but are not limited to Proximie, Explorer Surgical, Visual Lab 360, Kognito, Oxford Medical Simulation, Touch Surgery, Immersive Touch, Johnson & Johnson, and Medtronic. Commercial providers that do not have product-ready solutions or are currently still in development and/or lack relevant publicly available data will be excluded.

#### Snowball Searches

One-layer forward snowball searches (citation mining) and reverse snowballing (chain searching) will be applied to all included papers to capture related publications that may fall outside of the established search strategy. Any relevant review article (including systematic, scoping, literature, and narrative reviews) identified through any of the above searches will be retrieved and their reference lists screened for further potentially eligible records.

### Study Eligibility

Study eligibility criteria are defined according to the Population, Concept, and Context framework [38] and are presented in Table 2. Publications will only be considered if they are dated within 5 years of their original publication date to ensure data is contemporary. Articles will also be excluded if they are not published in the English language, due to resource restrictions that prevent the translation of non-English articles.

**Table 2:** Study eligibility criteria.

Element	Inclusion criteria	Exclusion criteria
Population	Adult (>18 years) human population Any individual qualified to undertake an invasive procedure (eg, junior doctors, surgeons, physicians, consultants, radiologists, endoscopists, gastroenterologists, cardiologists, advanced nurse practitioners)	Individuals not qualified to undertake an invasive procedure (eg, medical students, undergraduates)
Concept	Discuss, report, and/or evaluate one or more methods for shared learning (ie, for training/education and feedback) Must utilize digital means for shared learning Quantitatively or qualitatively evaluate the method for shared learning	Focus on digital method(s) for shared learning that are solely aimed to be used in situ (eg, laparoscopic techniques that have a camera installed and broadcast inside the operating theater) Simple descriptive presentation of the method for shared learning
Context	Must be in the context of invasive procedures	N/A^a^

^a^N/A: not applicable.

### Study Selection

For records with common publication formats (ie, published a title, structured abstract, executive summary, or synopsis), a 2-stage screening process will be undertaken to assess records for inclusion against the study eligibility criteria.

Search results will be downloaded from their respective online databases, deduplicated, and uploaded to an online review manager (Rayyan) [39]. In a first step, 3 review authors (CH, MK, and JR) will independently (each review author will be blind to the screening choice of the others) screen the titles and abstracts, executive summaries, or synopses of the retrieved records, with 10% of records double-screened. Full texts of articles will be obtained from records meeting the inclusion criteria and from those where inclusion remains uncertain (eg, because of a lack of information from the abstract). In a second step, 2 reviewers will each screen half of the retrieved full texts independently to assess full eligibility. Duplicate assessment of eligibility will be performed on 10% of all full texts with further duplicate reviews in case of poor agreement (<80%).

It is anticipated that some potentially relevant records do not follow conventional publication formats (eg, tweets, opinion pieces, news articles). In this case, 2 reviewers will independently review the content of the record in full.

Discrepancies at any stage of the screening process will initially be discussed between the 2 review authors. A third independent reviewer (SP) will arbitrate where agreement on inclusion could not be reached, and input from the wider team will be sought where necessary.

### Data Extraction

Data extraction will be performed directly into a purposely designed electronic data extraction form (eg, Microsoft Access; Microsoft Corp). Details about (1) study and publication characteristics (eg, author, study design, funding, and sponsorship statements), (2) the method for shared learning (eg, purpose, type, operationalization, and modality), and (3) impact of methods for shared learning (eg, methodology of evaluating methods for shared learning and their results, limitations, and author recommendations) will be extracted. Additional items of interest for social media posts will be explored to capture further information on their content (eg, presence of a link to an external website). An initial data extraction form will be piloted with a small number of relevant articles (see Multimedia Appendix 3). The form will be iteratively refined to comprehensively capture all relevant detail emerging during the pilot.

One review author will extract data from all included studies, and a second reviewer will independently perform double data extraction for at least 10% of articles. Consistency in the approach to data extraction will be ensured through constant dialogue between the 2 reviewers.

### Data Analysis

Findings will be summarized in tables using descriptive statistics and in narrative form. Verbatim extracted data will be analyzed by 2 reviewers adhering to principles of thematic analysis [40]. Identified themes will be displayed in schematics. Any verbatim extracted data will be reviewed to identify barriers and benefits to the implementation of shared learning methods. Barriers will be considered factors that impede the implementation of methods for shared learning in clinical practice. Benefits will be considered those that enable implementation [41]. Two reviewers will code data as a barrier or benefit, whereby regular meetings will be held to discuss coding results, and senior authors will be involved where consensus is required. In case an automated dashboard vendor is used to identify social media posts, the content will undergo additional analysis using the software’s existing classification algorithms and analyses (eg, sentiment analysis).

## Results

This work was initiated in October 2021. Iterative refinements to the scoping review protocol and formalizing of methods were completed in January 2021. Targeted searches were conducted in December 2021 to inform the development of a comprehensive search strategy for electronic database searches. This strategy was iteratively developed for and tested in MEDLINE. The final search was applied to MEDLINE and Web of Science in March 2022 and yielded a total of 10,093 records. Identification of relevant articles is currently ongoing and is expected to be completed by December 2022. Study selection, data extraction and analysis, and drafting of the manuscript to report the results of this scoping review will be conducted throughout 2023. Open access peer-reviewed publication is expected in 2023. Any changes to the methods reported here will be documented and reported.

## Discussion

This scoping review will explore methods for the application of digital methods for training/education and feedback for shared learning in the context of invasive surgical procedures. This work is, to the authors’ knowledge, the first to (1) summarize the application of existing methods for shared learning, (2) examine the impact of their application, and (3) explore the benefits and barriers to their implementation in the context of surgery. This scoping review protocol outlines a total of 6 different approaches to identify relevant articles and data to comprehensively map currently available information on this topic.

The results will provide an investigation of contemporary methods, which will be of interest to health care professionals and methodologists wishing to adopt methods for shared learning in surgical practice. Crucially, this work will contribute to ongoing research that aims to optimize safe and transparent innovation by promoting the sharing of incremental case-by-case learning among surgeons performing new procedures. Knowledge sharing in surgical innovation has not yet received much research attention, and there may be additional challenges that need to be considered. For example, surgeon innovators may be reluctant to share ideas and might show hesitancy in light of potential impacts on confidentiality or reputation. There is currently no evidence that demonstrates the underlying mechanisms that may impact surgeon innovators’ behavior toward sharing learning. This is an important avenue for future research requiring further exploration in qualitative work.

The findings from this scoping review will provide an initial step to inform the development of strategies to improve the efficiency and effectiveness of disseminating knowledge and information about novel procedures and devices. Essential to this ongoing work is the codevelopment of a real-time electronic platform that aims to collect, analyze, and feedback data about novel procedures and devices. Such an electronic platform will host a range of evidence-based approaches to safe and transparent surgical innovation that can facilitate the standardized collection and sharing of information and knowledge about novel procedures and devices [2,4]. Ultimately, enhancing shared learning in this way will reduce the risk of avoidable patient harm and streamline the evaluation of early-phase invasive procedures and devices.

This work will adhere to a robust methodology following the recommended standards for conducting scoping reviews [26,28,29]. This will ensure transparency and reduced risk of bias. Common limitations of scoping reviews, which also apply to our work, should be noted. Searches will be restricted to the English language, which limits our ability to summarize and examine findings from methods for shared learning in non–English-speaking contexts. Identifying surgical innovation and related information is hindered by poor reporting and informal dissemination. This work will intentionally address this challenge through an extensive search, but this may still not be sufficient to exhaustively capture all existing work using literature synthesis methods. Electronic database searches will be limited to 2 databases. Expert advice was sought, and it is expected that most of the relevant information on shared learning is included in these databases. However, there is a possibility that additional information of interest may be missed.

In conclusion, this scoping review will enhance our knowledge about the application of contemporary digital methods for training/education and feedback for shared learning in the context of invasive surgical procedures. This work is vital to help inform the development of novel methods to optimize shared learning in surgical innovation through the integration of findings into an electronic platform for real-time reporting and sharing of outcomes related to surgical innovation.

## Appendix

Multimedia Appendix 1 PRISMA-ScR (Preferred Reporting Items for Systematic Reviews and Meta-Analyses extension for scoping reviews) checklist.

Multimedia Appendix 2 Search strategy for database searches.

Multimedia Appendix 3 Initial list of data items to extract from eligible articles.

## Abbreviations

BRC: Biomedical Research Centre
IDEAL: Idea, Development, Exploration, Assessment, Long-term study
NIHR: National Institute for Health and Care Research
PRISMA: Preferred Reporting Items for Systematic Reviews and Meta-Analyses
PRISMA-ScR: PRISMA extension for scoping reviews

## Data Availability

This review will not generate any new data. All extracted data and results of the analyses will be made available as supplementary material in the publication of the review results.
