# Supplementary material for: Shared Learning Utilizing Digital Methods in Surgery to Enhance Transparency in Surgical Innovation: Protocol for a Scoping Review
Source: JMIR Res Protoc. 2022 Sep 8;11(9):e37544. doi: 10.2196/37544 (PMC9501681; doi:10.2196/37544)
Supplement: Multimedia Appendix 2 [file resprot_v11i9e37544_app2.pdf]

Multimedia Appendix to manuscript titled: Shared learning utilizing digital methods in surgery to enhance transparency in surgical innovation: A scoping review protocol

Search strategies for electronic database searches in Ovid (Medline) and Web of Science

**MEDLINE**
**Surgical staff (Population: criterion i,ii)**
exp Physicians/<br>exp Personnel, Hospital/<br>exp Medical Staff/<br>Health Personnel/<br>Consultants/<br>(surgeon$ OR clinician$ OR doctor$ OR physician$).tw
AND
**Feedback / Shared Learning (Intervention: criterion i)**
exp decision support techniques/<br>Knowledge of results.tw<br>Feedback, sensory<br>Biofeedback.tw<br>Knowledge/<br>Health Knowledge, Attitudes, Practice/<br>Information Dissemination/<br>(knowledge OR learn*) adj3 (utiliz* OR utilis* OR exchang* OR manag* OR sharing OR shared OR transfer* OR disseminat* OR distribut* OR mobilis* OR mobiliz* OR diffuse*).mp.<br>(multi-profession* OR multi-department* OR multi-institution* OR multi-occupation* OR inter-profession* OR inter-department* OR inter-occupation* OR inter-institution* OR peer OR peer to peer OR peer-to-peer OR peer-assisted OR cooperat* OR co-operat* OR collaborat* OR shared OR sharing OR joint OR team OR partnership) adj3 (work* OR learn* OR educat* OR communicat* OR disseminat*).tw<br>Collaboration.tw<br>Cooperative behaviour.tw<br>Formative Feedback/<br>Formative Feedback.tw<br>Learning/<br>Learning.tw<br>Feedback/<br>Feedback.tw<br>Shared learning.tw<br>Proctoring.tw<br>education, professional/<br>Educational Technology/<br>Health Education/<br>Education, Distance/
AND
**Digital Methods**
Digital Technology/<br>Electronics/

Robotics/<br>Telemedicine.tw<br>Teleproctoring.tw<br>Exp Computer simulation/<br>Remote sensing technology/<br>Telemetry/<br>Telemetry.tw<br>Informatics/<br>Informatics.tw<br>Real time monitor*.tw.<br>Computer-Assisted Instruction/<br>Computer Simulation/<br>Patient Simulation/<br>exp Models, Anatomic/<br>exp Telemedicine/<br>Simulation training/<br>Simulation.tw<br>Simulation training.tw<br>exp Videoconferencing/<br>(augmented OR augment* OR virtual OR visual OR digital* OR online OR on-line OR computer supported OR technology based OR technology enhanced OR web-based OR web based) adj3 (learning OR educ*)<br>(E-learn* OR e_learn* OR elearn* OR mlearn* OR m_learn* OR m-learn*).tw
AND
**Invasive procedure (Intervention: criterion ii)**
invasive.tw.<br>incision.tw.<br>cut.tw.<br>percutaneous.tw.<br>puncture.tw.<br>(natural adj1 orifice).tw.<br>endoscop*.tw.<br>colonoscop*.tw.<br>gastroscop*.tw.<br>laparoscop*.tw.<br>catheter.tw.<br>scalpel.tw.<br>surgery.tw.<br>surgical.tw.<br>operat*.tw.<br>interventional.tw.<br>device*.tw.<br>implant*.tw.<br>prosthe*.tw.<br>robotic*.tw.<br>exp Specialties, Surgical/<br>exp Endoscopy/<br>Radiology, Interventional/<br>exp Surgical Procedures, Operative/<br>exp "prostheses and implants"/

or/1-25<br>humans/<br>animals/<br>27 not 28<br>26 and 29
**Results: 3,402**

**WEB OF SCIENCE**
**Surgical staff (Population: criterion i,ii)**
TS=(doctor* OR surgeon* OR physician*)
AND
**Feedback / Shared Learning (Intervention: criterion i)**
TS=((Shar* NEAR/3 Learn*) OR feedback OR (information disseminat*) OR collaborat* OR (cooperative behaviour) OR (co-operative behaviour) OR (formative feedback) OR feedback) OR TS=((knowledge OR learning) NEAR/3 (utiliz* OR utilis* OR exchang* OR manag* OR sharing OR shared OR transfer* OR disseminat* OR distribut* OR mobilis* OR mobiliz* OR diffuse*)) OR TS=((multi-profession* OR multi-department* OR multi-institution* OR multi-occupation* OR inter-profession* OR inter-department* OR inter-occupation* OR inter-institution* OR peer OR "peer to peer" OR peer-to-peer OR peer-assisted OR cooperat* OR co-operat* OR collaborat* OR shared OR sharing OR joint OR team OR partnership) NEAR/3 (work* OR learn* OR education OR communicat* OR disseminat*)) OR<br>TS=(Collaboration OR (Cooperative behaviour) OR (Formative Feedback) OR Proctor*) OR<br>TS=(Proctor* OR Train* OR (simulat* NEAR/3 train*) OR (Health NEAR/0 Education))
AND
**Digital Methods**
TS=(Robotic* OR Telemed* OR Tele-med* OR Teleproctor* OR tele-proctor* OR Simulat* OR (patient simulat*) OR (augmented reality) OR (virtual reality) OR AR OR VR OR elearn* OR e-learn* OR mlearn*) OR<br>TS=((augmented OR augment* OR virtual OR visual OR digital* OR online OR on-line OR "computer supported" OR "technology based" OR "technology enhanced" OR web-based OR "web based") NEAR/3 (learning OR educ*)) OR<br>TS=(E-learn* OR e_learn* OR elearn* OR mlearn* OR m_learn* OR m-learn*)
AND
**Invasive procedure (Intervention)**
TS=(invasive OR incision OR cut OR percutaneous OR puncture OR (natural orifice) OR endoscop* OR colonscop* OR gastroscop* OR laparoscop* OR catheter OR scalpel or surgery OR surgical OR opera* OR interventional OR device* OR implant* OR prosthe* OR robotic*) OR WC=Surgery
**Results: 8,301**
