# Supplementary material for: Shared Learning Utilizing Digital Methods in Surgery to Enhance Transparency in Surgical Innovation: Protocol for a Scoping Review
Source: JMIR Res Protoc. 2022 Sep 8;11(9):e37544. doi: 10.2196/37544 (PMC9501681; doi:10.2196/37544)
Supplement: Multimedia Appendix 3 [file resprot_v11i9e37544_app3.pdf]

Multimedia Appendix to manuscript titled: Shared learning utilizing digital methods in surgery to enhance transparency in surgical innovation: A scoping review protocol

Initial list of data items for extracting information from eligible articles

1. Publication and study characteristics
    - Surname of author
    - Year of publication
    - Country of origin
    - Affiliated institutions (e.g. private/HE/hospital)
    - Subject field (e.g. surgical specialty)
    - Device or technique
    - Hospital setting
    - Publication type (e.g. protocol, peer-reviewed article, opinion piece, tweet)
    - Study type (if applicable) (e.g. RCT, cohort, cross-sectional)
    - Funding and sponsorship statements (e.g. industry/public)
    - Conflict of interest statements

2. Method for shared learning
    - Purpose of using shared learning (e.g. training, feedback, assessment of skill)
    - Details of the individuals intended to receive/receiving the training/feedback etc (e.g. surgeons, trainees)
    - How was the learning shared?
        - Any definitions
        - Were any models for shared learning used?
        - If yes, what were the key concepts?
    - Details about the feedback
        - Type of feedback (e.g. augmented, outcome, visual)
        - Methods and mechanisms
    - How was shared learning operationalized? (e.g. training, education)
        - Modality of education or training (e.g. courses / AR / VR / video / other)
        - Methods for providing feedback
        - Whether the education/training/feedback was provided in real-time
    - Author recommendations for next steps (e.g. if an intervention whether randomised controlled trial is now needed) (verbatim)

3. Impact of methods for shared learning (i.e. benefits and barriers)
    - Surgeon/user feedback (if assessed) (e.g. acceptability of the methods to surgeons, patients and hospital trusts (verbatim)
    - Was the shared learning method formally evaluated? was learning, performance and technique evaluated? (e.g. interviews, focus groups, survey)
        - How was it evaluated (qualitative, quantitative, or mixed-methods)
        - What was the method? (Interviews, focus groups, survey etc)
        - What was the outcome measured?
        - What are the results of the evaluation?
    - Details of any outcomes of evaluation of the methods' effectiveness (verbatim, data)
    - Were any limitations of the application of shared learning reported
    - Key conclusions made by authors (verbatim)
    - Additional comments of interest
